# CRISPRi-mediated silencing of the ABC transporter *Ms_5779* compromises cell envelope integrity and impairs intracellular host lipid exploitation

**DOI:** 10.3389/fmicb.2026.1835283

**Published:** 2026-06-09

**Authors:** Yuhao Song, Didi Ban, Rui Zhang, Naichao Diao, Jianming Li, Kun Shi, Fanli Zeng, Rui Du

**Affiliations:** 1 College of Animal Science and Technology, Jilin Agricultural University, Changchun, China; 2 College of Chinese Medicine Materials, Jilin Agricultural University, Changchun, China; 3 Jilin Province Sika Deer Efficient Breeding and Product Development Technology Engineering Research Center, Jilin Agricultural University, Changchun, China; 4 College of Agriculture, Yanbian University, Yanji, China

**Keywords:** ABC transporter, CRISPRi, drug resistance, *Mycobacterium smegmatis*, *Rv0820/Ms_5779*

## Abstract

**Background:**

Multidrug-resistant *Mycobacterium tuberculosis* (MDR-TB) poses a severe global health threat, yet the contribution of cell envelope barriers to intrinsic drug tolerance remains poorly understood. The ABC protein *Rv0820* is conserved across mycobacteria but functionally uncharacterized, presenting a structural paradox: it lacks canonical transmembrane helices, making conventional efflux pump function mechanistically implausible.

**Methods:**

Using CRISPR interference (CRISPRi), we silenced *Ms_5779*—the *Mycobacterium smegmatis (M. smegmatis)* ortholog of *Rv0820*—to two graded depths (81% and 69% repression) and subjected the resulting strains to systematic phenotypic interrogation spanning drug susceptibility, cell envelope permeability, biofilm formation, multi-stress tolerance, and macrophage infection.

**Results:**

*Ms_5779* silencing did not affect growth kinetics but markedly disrupted cell envelope integrity, as evidenced by impaired biofilm formation, dissipated membrane potential, and increased ethidium bromide permeability. Silenced strains exhibited broad-spectrum drug hypersusceptibility across six antibiotic classes, with the most striking effect being a 32-fold reduction in norfloxacin MIC, alongside heightened sensitivity to oxidative, acidic, and thermal stress. In a RAW264.7 macrophage infection model, *Ms_5779* silencing attenuated intracellular survival, impaired host cholesterol exploitation and foamy macrophage induction, and reduced apoptosis induction.

**Conclusion:**

Collectively, these findings strongly suggest that *Ms_5779*/Rv0820 is a critical functional contributor indispensable for the maintenance of cell envelope integrity, presenting a distinct mechanism from a conventional efflux pump. Targeting this essential uncharacterized ABC protein would simultaneously compromise the drug permeability barrier and the intracellular niche, nominating *Rv0820* as a mechanistically distinct therapeutic target for anti-tuberculosis drug discovery.

## Introduction

*Tuberculosis* (TB) has accompanied humanity for millennia, yet in 2024 it still claimed an estimated 1.23 million lives and afflicted 10.7 million people worldwide, making *Mycobacterium tuberculosis* (Mtb) the leading cause of death from a single infectious agent. According to the WHO Global Tuberculosis Report 2025 (World Health Organization, 2025), Multidrug-resistant tuberculosis (MDR/RR-TB) now affects 3.2% of new cases and 16% of previously treated patients, with approximately 390,000 incident rifampicin-resistant cases in 2024 alone. Most alarming is the trajectory of pre-XDR-TB (Extensively drug-resistant tuberculosis, strains combining MDR/RR-TB with fluoroquinolone resistance), which already accounts for 19% of the MDR/RR-TB burden globally. Fluoroquinolones are the backbone of second-line regimens, making their declining efficacy a critical challenge. Therefore, identifying novel drug targets and dissecting drug tolerance mechanisms is urgently required.

The molecular basis of Mtb drug resistance extends beyond canonical target-gene mutations (rpoB, katG/inhA, gyrA/gyrB), which alone cannot fully explain low-level or heteroresistance in clinical isolates (Alvarado-Peña et al., 2025; Chimal-Muñoz et al., 2025). This gap underscores active drug efflux as a vital complementary resistance mechanism (Remm et al., 2022; Datta et al., 2024). By transiently keeping intracellular drug concentrations sub-lethal, efflux pumps serve as a first-line defense, buying bacteria time to acquire high-level resistance mutations (Rodrigues et al., 2020). Furthermore, their role extends beyond mere drug tolerance: these pumps are robustly upregulated during antibiotic exposure (Chauhan et al., 2025; Meng et al., 2025) and directly contribute to virulence, biofilm architecture, and macrophage survival (Lake et al., 2023). Consequently, efflux pumps act as dynamic participants in the pathogen’s broader adaptation to host environments.

Among the five efflux pump superfamilies in mycobacteria, the ATP-driven ABC family is the largest yet most functionally enigmatic (Elshobary et al., 2025; Tursun et al., 2025). While the Mtb genome encodes at least 37 ABC transporters (de Cassio Barreto Oliveira and Balan, 2020; Soni et al., 2020; Mandal et al., 2021), only a small handful have been well-characterized (Wang et al., 2024; Adhikary et al., 2023; Adhikary et al., 2022; Singh and Dutta, 2024). The remaining ~30 predicted members remain functionally mysterious (Soni et al., 2020; Sharma et al., 2023). Because these transporters can couple ATP hydrolysis to drive efflux against steep concentration gradients, shifting from bioinformatic predictions to the experimental interrogation of these uncharacterized members is critical to closing this knowledge gap.

*Rv0820* is one such uncharacterized locus, and it presents an immediate structural paradox. Sequence analysis places it within the ABC superfamily, predicting an efflux function, yet TMHMM topology analysis finds no canonical transmembrane helices, and SignalP-5.0 assigns a signal peptide probability that is extremely low. A transporter without a membrane-spanning domain cannot, by definition, translocate substrates across a lipid bilayer in the conventional sense. This raises a question that is both mechanistically interesting and therapeutically relevant: what does *Rv0820* actually do, and why does its disruption matter? Rv0820 has been annotated in the literature as PhoT, a homolog of the ATP-binding subunit (PstB) of the phosphate-specific transport (Pst) system. The Pst system is a high-affinity ABC importer responsible for inorganic phosphate (Pi) acquisition under Pi-limiting conditions, and is conserved across mycobacteria (Braibant et al., 1996, 2000). The protein’s high sequence conservation with *Ms_5779* in the rapidly growing, BSL-1 surrogate *M. smegmatis* mc^2^-155 (Shiloh and Champion, 2010; Sparks et al., 2023) provides a tractable experimental entry point. *M. smegmatis* has been repeatedly validated as a faithful model for mycobacterial cell wall biology and drug resistance mechanisms, and the conservation of *Ms_5779*/*Rv0820* across the mycobacterial genus argues strongly that whatever function this protein serves, it is ancestral and likely essential to the mycobacterial lifestyle.

Interrogating a gene of unknown function in Mtb is technically demanding: homologous recombination is inefficient, and disrupting a conditionally essential locus can be lethal, precluding the construction of clean knockouts. CRISPRi sidesteps both problems. By directing a catalytically dead Cas9 (dCas9) to a target promoter or early coding sequence via a single guide RNA (sgRNA), CRISPRi sterically blocks RNA polymerase without cleaving DNA, achieving conditional, and reversible transcriptional repression (Wong and Rock, 2021; Zhang et al., 2021). Critically, the depth of silencing can be titrated by choosing sgRNAs that target different positions within the gene, enabling dose–response analysis that is impossible with conventional knockouts (Bosch et al., 2021; Li et al., 2022). The approach has also been deployed in macrophage infection models (Cheung et al., 2022; Liu et al., 2025). Particularly relevant to the present study, CRISPRi silencing of cell wall biosynthesis genes in *M. smegmatis*, including mmpL3 (Yuliani et al., 2024) and cwlM (Yang et al., 2025), has directly linked cell wall integrity to antibiotic susceptibility, establishing a conceptual framework that we apply here to *Ms_5779*.

Here, we address the *Rv0820* paradox experimentally. Using a two-plasmid CRISPRi system in *M. smegmatis* mc^2^-155, we silenced *Ms_5779* to two graded depths and subjected the resulting strains to systematic phenotypic interrogation spanning drug susceptibility, cell wall permeability, biofilm formation, multi-stress tolerance, and macrophage infection. The data converge on a coherent model: *Ms_5779* is an essential functional component as a cell wall structural determinant whose integrity is required for the permeability barrier that underlies broad-spectrum drug tolerance, stress resistance, and intracellular pathogenesis. The fluoroquinolone hypersusceptibility phenotype (up to 32-fold MIC reduction) is particularly striking. Together, these findings recast *Rv0820* as a mechanistically tractable and therapeutically relevant target, and demonstrate the power of graded CRISPRi silencing for dissecting the function of structurally atypical, uncharacterized mycobacterial genes.

## Methods

### Bacterial strains, plasmids, and culture conditions

*Mycobacterium smegmatis* mc^2^-155 (ATCC 700084), maintained in our laboratory collection, was used as the model organism throughout this study. Bacteria were routinely cultured in Middlebrook 7H9 broth supplemented with 10% ADC enrichment (BD Difco) and 0.05% Tween-80, or on Middlebrook 7H10 agar supplemented with 10% OADC enrichment, at 37 °C with shaking at 200 rpm. Escherichia coli DH5α was used for plasmid propagation and was cultured in Luria-Bertani (LB) broth or agar at 37 °C with shaking at 220 rpm. All strains were stored as glycerol stocks (20% v/v glycerol) at −80 °C. The CRISPRi plasmids pRH2502 (constitutive dCas9 expression, kanamycin resistance) and pRH2521 (sgRNA expression scaffold, hygromycin resistance) were obtained from Addgene and have been described previously (Wong and Rock, 2021). Antibiotic concentrations used for selection were: kanamycin sulfate, 25 μg/mL (for *M. smegmatis*) or 50 μg/mL (for *E. coli*); hygromycin B, 50 μg/mL (for *M. smegmatis*) or 150 μg/mL (for *E. coli*).

### Bioinformatic analysis

The amino acid sequences of Mycobacterium were retrieved from the Mycobrowser database.^1^ Multiple sequence alignment was performed using ClustalW^2^ with default parameters. The resulting alignment was visualized and rendered using ESPript 3.0,^3^ with identical residues highlighted in red boxes and conserved residues shown in red text. Conserved domains and signature motifs characteristic of ABC transporters were identified using the NCBI Conserved Domain Database (CDD) and Pfam database. Physicochemical properties of both proteins were computed using the ProtParam tool on the ExPASy proteomics server,^4^ including molecular weight (MW), theoretical isoelectric point (pI), instability index, aliphatic index, and grand average of hydropathicity (GRAVY). Signal peptide and lipoprotein signal peptide probabilities were predicted using SignalP-5.0^5^ with the Gram-positive organism setting. Transmembrane helix topology was predicted using TMHMM Server v2.0.^6^

### CRISPRi system construction

sgRNAs targeting the *Ms_5779* coding sequence were designed using Benchling software,^7^ selecting 20-nt protospacer sequences on the non-template strand within the first 200 bp of the coding region, immediately upstream of an NGG PAM sequence. Two sgRNAs were selected based on high on-target scores (Doench 2016 rule set) and minimal predicted off-target activity: Ms_5779 + 57 (targeting position +57 relative to the transcription start site) and *Ms_5779* + 162 (targeting position +162). A non-targeting control sgRNA (sgRNA-NC) was constructed using a 20-nt protospacer sequence derived from the SPUD sequence of potato (*Solanum tuberosum*), which has no homology to the *M. smegmatis* genome (Xiao, 2018). The corresponding oligonucleotides for all three sgRNAs (with BbsI-compatible overhangs) were synthesized commercially (Shanghai Sangon Biotech Company). Oligonucleotide pairs were annealed in NEB Buffer 2.1 (New England Biolabs) by heating to 95 °C for 5 min followed by gradual cooling to room temperature at a rate of 0.1 °C/s in a thermocycler. The pRH2521 plasmid was linearized by BbsI restriction digestion (37 °C, 1 h), gel-purified, and the annealed sgRNA duplexes were ligated into the linearized vector using T4 DNA Ligase (EL0011, Thermo Fisher, USA) according to the manufacturer’s instructions. Recombinant plasmids were transformed into chemically competent *E. coli* DH5α cells, and positive colonies were screened by colony PCR.

*Mycobacterium smegmatis* mc^2^-155 electrocompetent cells were prepared as follows: late-log phase cultures (OD₆₀₀ = 0.8–1.0) were harvested by centrifugation (3000 × *g*, 10 min, 4 °C), washed three times with ice-cold 10% glycerol, and resuspended in 10% glycerol at a final concentration of approximately 10^10^ cells/mL. Electroporation was performed using 100 μL of competent cells mixed with 1–2 μg of plasmid DNA in a pre-chilled 2 mm electroporation cuvette (2.5 kV, 1,000 *Ω*, 25 μF; Bio-Rad Gene Pulser Xcell). Immediately after electroporation, cells were resuspended in 1 mL of pre-warmed 7H9 broth and allowed to recover at 37 °C with shaking (200 rpm) for 3 h before plating on selective 7H10 agar. Transformant colonies were selected after 3–5 days of incubation at 37 °C. The pRH2502 plasmid was first electroporated into wild-type *M. smegmatis* mc^2^-155 to generate the Ms-dCas9 strain; the sgRNA-expressing plasmids (pRH2521-*Ms_5779* + 57, pRH2521-*Ms_5779* + 162, and pRH2521-sgRNA-NC) were subsequently electroporated into Ms-dCas9 to generate the final experimental and control strains.

### RNA extraction and RT-qPCR

Total RNA was extracted from mid-log phase cultures (OD₆₀₀ = 0.4–0.8). Bacterial cells were harvested by centrifugation (3000 × *g*, 10 min, 4 °C), resuspended in lysis buffer, and disrupted by bead-beating (using 0.1 mm zirconia-silica beads, 3 cycles of 60 s at 60 Hz, with 1 min intervals on ice) using a Scientz-48 high-throughput tissue homogenizer (Scientz Biotechnology, Ningbo, China). RNA was purified using the E.Z.N.A.® Bacterial RNA Kit (R6950-00, Omega Biotek, USA) according to the manufacturer’s instructions, including an on-column DNase I digestion step to eliminate genomic DNA contamination. RNA quality and quantity were assessed by measuring the A₂₆₀/A₂₈₀ ratio (acceptable range: 1.8–2.1), and RNA integrity was confirmed by 1% agarose gel electrophoresis. cDNA was synthesized from 500 ng of total RNA using the PrimeScript RT Reagent Kit with gDNA Eraser (RR047Q, TaKaRa, China) according to the manufacturer’s protocol (42 °C, 15 min; 85 °C, 5 s). RT-qPCR was performed using TB Green Premix Ex Taq II (RR091Q, TaKaRa, China) on a QuantStudio (Thermo Fisher, USA) with the following cycling conditions: 95 °C for 30 s (initial denaturation); 40 cycles of 95 °C for 5 s and 60 °C for 30 s; followed by a melt curve analysis (65 °C–95 °C, 0.5 °C increments). All reactions were performed in technical quadruplicate in 20 μL volumes. Primer amplification efficiencies were validated by standard curve analysis (serial 5-fold dilutions of cDNA; acceptable efficiency range: 90–110%, R^2^ ≥ 0.99). Relative gene expression was calculated using the 2^−ΔΔCt^ method (Livak and Schmittgen, 2001).

### Growth curve analysis

Bacterial cultures were grown to mid-log phase (OD₆₀₀ = 0.4–0.6), adjusted to a standardized OD₆₀₀ of 0.05 in fresh 7H9 broth, and inoculated at 1% (v/v) into 50 mL of 7H9 broth in 250 mL baffled Erlenmeyer flasks. Cultures were incubated at 37 °C with shaking at 110 rpm. OD₆₀₀ was continuously monitored over a 120-h period. Three independent biological replicates were performed for each strain, and growth curves were plotted as mean OD₆₀₀ ± SEM.

### Biofilm formation assay

Bacteria were grown to mid-log phase, washed three times with PBS to remove residual medium components, and adjusted to OD₆₀₀ = 0.1 in fresh 7H9 broth. Cultures were inoculated at 10% (v/v) into 12-well plates containing 2 mL of 7H9 broth per well and incubated at 37 °C without shaking for 5 days to allow biofilm formation at the air–liquid interface. After incubation, the culture medium and non-adherent planktonic cells were carefully aspirated. Biofilms were gently washed three times with sterile PBS to remove loosely attached cells, then fixed with 100% methanol for 15 min at room temperature. After air-drying, biofilms were stained with 1% (w/v) crystal violet solution for 20 min at room temperature, washed three times with distilled water to remove excess stain, and air-dried completely. Bound crystal violet was solubilized by adding 1 mL of 33% (v/v) glacial acetic acid per well and incubating for 30 min at room temperature with gentle agitation (Recht and Kolter, 2001). Absorbance was measured at OD₅₆₀ using a microplate reader.

### Minimum inhibitory concentration (MIC) determination

MICs were determined by the broth microdilution method in sterile 96-well flat-bottom plates, following Clinical and Laboratory Standards Institute (CLSI) guidelines M24-Ed3 for susceptibility testing of mycobacteria, adapted for *M. smegmatis*. Drug stock solutions were prepared as follows: rifampicin (RIF) and norfloxacin (NOR) were dissolved in dimethyl sulfoxide (DMSO); isoniazid (INH), streptomycin (SM), capreomycin (CPM), and moxifloxacin (MXF) were dissolved in sterile distilled water. All stock solutions were prepared at 1,000× the highest test concentration and stored at −20 °C. Two-fold serial dilutions of each drug were prepared in 7H9 broth in a total volume of 100 μL per well, covering a concentration range of 0.0078–64 μg/mL. The final DMSO concentration in all wells did not exceed 1% (v/v). Bacterial inocula were prepared by adjusting mid-log phase cultures to a 0.5 McFarland standard (approximately 1 × 10^8^ CFU/mL) in 7H9 broth, then diluting 1:200 to yield a final inoculum of approximately 5 × 10^5^ CFU/mL in each well. Each plate included a growth control (bacteria without drug), a sterility control (medium without bacteria), and a DMSO vehicle control (bacteria with 1% DMSO, no drug). Plates were sealed with adhesive film and incubated at 37 °C for 7 days without shaking. The MIC was defined as the lowest drug concentration that completely inhibited visible bacterial growth as determined by unaided visual inspection.

### Ethidium bromide accumulation assay

Bacterial cultures were grown to mid-log phase, harvested by centrifugation (3000 × *g*, 10 min), washed twice with PBS, and resuspended in PBS to OD₆₀₀ = 0.4. Bacterial suspensions (200 μL per well) were transferred to black flat-bottom 96-well plates (Corning). Ethidium bromide (EB) was added to a final concentration of 1 μg/mL. Fluorescence intensity (excitation 545 nm, emission 590 nm) was measured at 5-min intervals for 60 min at room temperature (25 °C) in the dark. Three independent biological replicates were performed.

### Membrane potential measurement

Bacterial cultures were prepared as described in Ethidium bromide accumulation assay and resuspended in PBS to OD₆₀₀ = 0.4. Rhodamine 123 (IR1800; Beijing Solarbio Science & Technology Co., Ltd., China), a membrane potential-sensitive fluorescent dye that accumulates in cells with intact membrane potential, was added to bacterial suspensions (200 μL per well in black 96-well plates) at a final concentration of 20 μM and incubated at 37 °C for 30 min in the dark to allow equilibration. Fluorescence intensity (excitation 480 nm, emission 530 nm) was measured at 1-min intervals for 15 min at 37 °C. Three independent biological replicates were performed.

### Stress survival assays

For all stress assays, bacterial cultures were grown to mid-log phase (OD₆₀₀ = 0.4–0.6), harvested by centrifugation, washed twice with PBS, and resuspended to OD₆₀₀ = 0.1 (approximately 1 × 10^7^ CFU/mL) in the appropriate buffer or medium.

Oxidative stress (disk diffusion assay): Bacterial suspensions (200 μL, approximately 2 × 10^6^ CFU) were spread uniformly onto 7H10 agar plates using a sterile cotton swab. Sterile filter paper disks (6 mm diameter; Whatman) saturated with 20 μL of 1% (v/v) H₂O₂ were placed on the bacterial lawn. Plates were incubated at 37 °C for 2–3 days, and inhibition zone diameters (including the disk diameter) were measured using a digital caliper. Three independent experiments were performed.

Acid stress survival assay: Bacterial suspensions were resuspended in Middlebrook 7H9 broth adjusted to pH 3.0 with HCl (verified by pH meter immediately before use). The pH 3.0 condition was selected to model the highly acidic environment of the phagolysosome, which can reach pH values as low as 4.5–5.0 in activated macrophages and potentially lower in certain cellular compartments. While pH 3.0 is more extreme than typical phagolysosomal conditions, it provides a stringent *in vitro* stress condition that allows discrimination between strains with subtle differences in acid tolerance, consistent with approaches used in previous mycobacterial stress studies(Vandal et al., 2008; Paroha et al., 2018). Cultures were incubated at 37 °C with shaking at 200 rpm. At 0, 6, 12, 24, and 36 h, 100 μL aliquots were withdrawn, serially diluted 10-fold in PBS (10^−1^ to 10^−6^), and 10 μL of each dilution was spotted onto 7H10 agar plates. Bacteria resuspended in 7H9 broth at pH 7.0 served as the negative control. Colony-forming units (CFU) were counted after 3–4 days of incubation at 37 °C, and survival rates were expressed as the percentage of CFU relative to the *t* = 0 time point. Three independent biological replicates were performed.

Heat stress survival assay: Bacterial suspensions (1 mL in PBS) were transferred to 1.5 mL microcentrifuge tubes and incubated at 53 °C in a thermostatic water bath. At 0, 1, and 2 h, 100 μL aliquots were withdrawn, serially diluted 10-fold in PBS (10^−1^ to 10^−5^), and 10 μL of each dilution was spotted onto 7H10 agar plates. Bacteria maintained at 37 °C in PBS served as the negative control. CFU were counted after 3–4 days at 37 °C, and survival rates were expressed as the percentage of CFU relative to the t = 0 time point. Three independent biological replicates were performed.

### Macrophage infection assays

RAW264.7 murine macrophages (ATCC TIB-71) were maintained in Dulbecco’s Modified Eagle’s Medium (DMEM; 11965118, Gibco, USA) supplemented with 10% heat-inactivated fetal bovine serum (A5669701, Gibco, Australia) and 1% penicillin–streptomycin at 37 °C in a humidified atmosphere of 5% CO₂. For infection experiments, macrophages were seeded at 5 × 10^5^ cells per well in 24-well plates and allowed to adhere and equilibrate for 24 h prior to infection. Penicillin–streptomycin was omitted from the culture medium 12 h before infection to avoid interference with bacterial viability.

Bacterial cultures were grown to mid-log phase, harvested by centrifugation, washed twice with PBS, and resuspended in antibiotic-free DMEM. Bacterial concentrations were determined by measuring OD₆₀₀ and confirmed by CFU counting on 7H10 agar. Macrophages were infected at a multiplicity of infection (MOI) of 10 bacteria per macrophage for 2 h at 37 °C in 5% CO₂ (Cheung et al., 2022). After infection, cells were washed three times with warm PBS to remove extracellular bacteria, and fresh DMEM containing gentamicin (50 μg/mL) was added to eliminate any remaining extracellular bacteria. Cells were incubated for an additional 2 h. Gentamicin was selected because it is a standard membrane-impermeable aminoglycoside that effectively eliminates extracellular mycobacteria without penetrating the host cells or inducing macrophage cytotoxicity. Furthermore, the bactericidal efficacy of gentamicin against all *M. smegmatis* strains used in this study (Ms_Ctrl, Ms_5779+57, and Ms_5779+162) was experimentally verified prior to infection, with a determined minimum inhibitory concentration (MIC) of 2 ug/mL (Hershko et al., 2023), which is well below the 100 ug/mL treatment concentration. Then washed twice with PBS, and fresh antibiotic-free DMEM was added. This time point was designated as 0 h post-infection for subsequent measurements. To directly quantify intracellular bacterial survival, infected RAW264.7 macrophages were processed for colony-forming unit (CFU) enumeration at 0, and 24 h post-infection. At each designated time point, the culture medium was removed, and the macrophage monolayers were thoroughly washed three times with sterile PBS to eliminate any residual extracellular bacteria. Subsequently, the cells were lysed by adding 0.1% (v/v) Triton X-100 in PBS and incubated for 10 min at room temperature. The resulting cell lysates were serially diluted (10-fold) in sterile PBS, and appropriate dilutions were spread onto Middlebrook 7H10 agar plates. Following an incubation period of 3–4 days at 37 °C, visible colonies were counted. The intracellular viable bacterial load was calculated and expressed as Log10 CFU/mL. All experiments were performed in biological triplicate.

Cell viability (CCK-8 assay): At 0, 2, 4, 8, 12, and 16 h post-infection, 10 μL of Cell Counting Kit-8 (CCK-8) reagent (CA1212, Beijing Solarbio Science & Technology Co., Ltd., China) was added to each well containing 100 μL of medium, and plates were incubated at 37 °C for 2 h. Absorbance was measured at 450 nm. Cell viability was expressed as a percentage relative to uninfected control wells.

Nitric oxide (NO) measurement: At each time point, NO production was quantified using Nitric Oxide Assay Kit (S0021S, Beyotime, China) according to the manufacturer’s instructions. Briefly, 50 μL of supernatant was mixed with equal volumes of Griess Reagent I and Griess Reagent II, incubated for 10 min at room temperature, and absorbance was measured at 540 nm. NO concentrations were calculated from a sodium nitrite standard curve.

Cholesterol measurement: Intracellular cholesterol content was determined at 0, 12, 24, and 36 h post-infection using Total Cholesterol Assay Kit (S0211S, Beyotime, China) according to the manufacturer’s instructions. Cells were lysed in the provided lysis buffer, and cholesterol was quantified colorimetrically at 570 nm. Cholesterol concentrations were calculated from standard curve.

Oil Red O staining: At 24 h post-infection, cells were washed with PBS, fixed with 4% paraformaldehyde (PFA) in PBS for 20 min at room temperature, washed twice with PBS, and rinsed with 60% isopropanol for 5 min. Cells were then stained with freshly prepared Oil Red O working solution for 20 min at room temperature, washed three times with distilled water, and counterstained with Mayer’s hematoxylin for 1 min. After washing, cells were examined and photographed under a light microscope. For quantitative analysis, nuclei in the blue-dominant channel were counted using Otsu thresholding and watershed segmentation. Lipid droplet signal was quantified as the integrated optical density (IntDen) of the red channel within an HSV color mask. The signal intensity per cell was first calculated as the mean of the ratios of total IntDen to the total number of cells across all ROIs, and then normalized to that of Ms_Ctrl-infected cells to be expressed as a percentage of the control.

Apoptosis detection by flow cytometry: At 16 h post-infection, cells were detached using a cell scraper, washed twice with cold PBS, and resuspended in 1× Annexin V binding buffer. Cells were stained with Annexin V-FITC and propidium iodide (PI) using the Annexin V-FITC/PI Apoptosis Detection Kit (ab14085, abcam, USA) according to the manufacturer’s instructions, and incubated for 15 min at room temperature in the dark. A minimum of 10,000 events per sample were acquired on a BD FACSVerse flow cytometer (BD Biosciences). Data were analyzed using FlowJo software (version 10.8; BD Biosciences). Gating strategy: live cells were first gated based on forward scatter (FSC) and side scatter (SSC) to exclude debris; apoptotic populations were then defined as early apoptotic (Q3, Annexin V^+^/PI^−^), late apoptotic (Q2, Annexin V^+^/PI^+^), and necrotic (Q1, Annexin V^−^/PI^+^).

### Statistical analysis

All experiments were performed in biological triplicate unless otherwise stated. Data are presented as mean ± standard error of the mean (SEM). For comparisons among three or more groups, one-way analysis of variance (ANOVA) followed by Tukey’s honestly significant difference (HSD) *post-hoc* test was used. For pairwise comparisons between two groups, Student’s two-tailed unpaired *t*-test was applied. All statistical analyses were performed using IBM SPSS Statistics version 22.0 (IBM Corp., Armonk, NY, USA). Graphs were generated using GraphPad Prism version 7.0 (GraphPad Software, San Diego, CA, USA). A *p*-value of < 0.05 was considered statistically significant and is indicated as follows: *p < 0.05, **p < 0.01, ***p < 0.001, ****p < 0.0001.

## Results

### Bioinformatic analysis predicts *Rv0820* and *Ms_5779* as conserved cell wall-associated ABC transporters lacking canonical transmembrane domains

**Figure 1:**
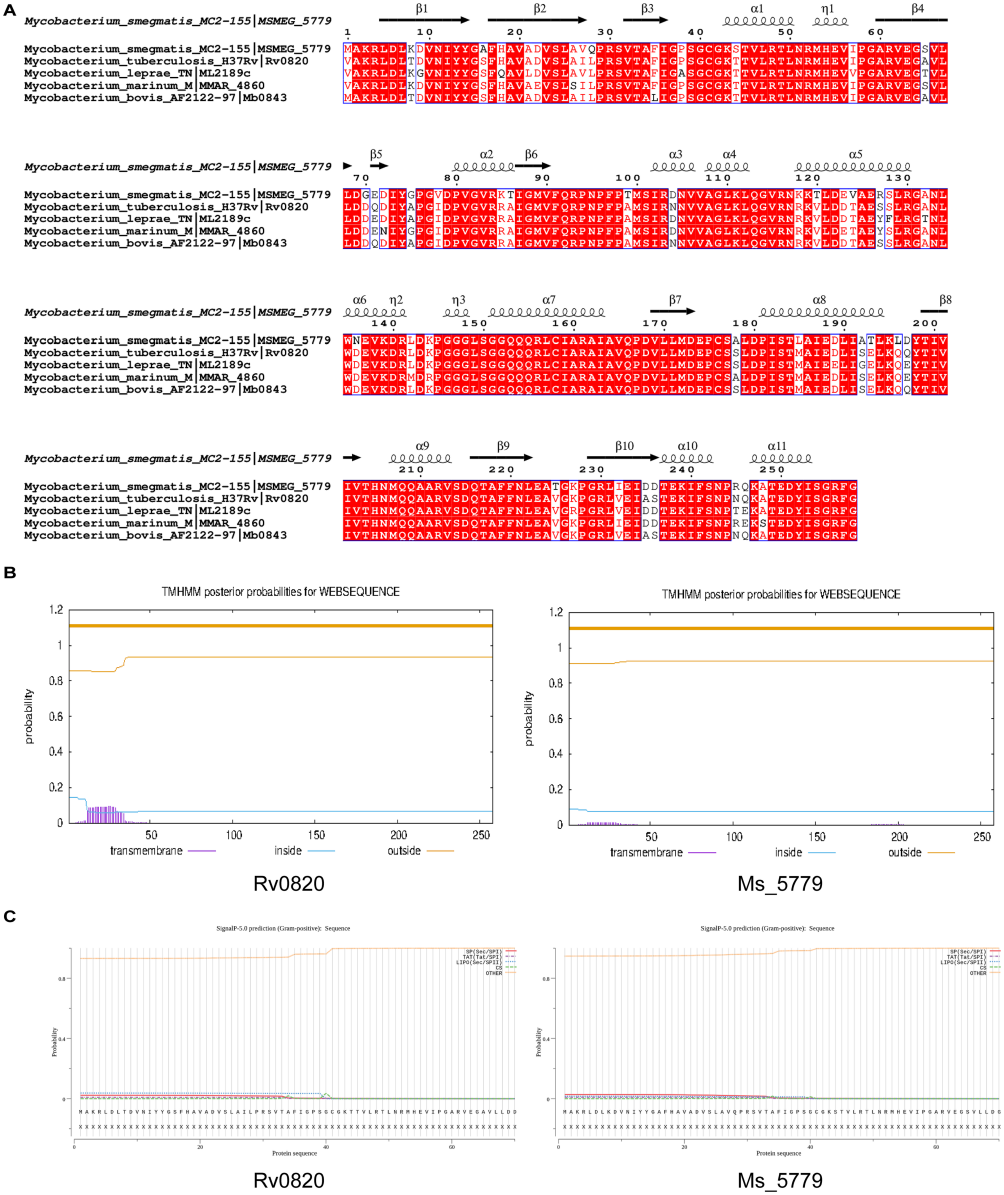
Bioinformatic analysis of *Rv0820* and its mycobacterial orthologs. **(A)** Multiple sequence alignment of *Rv0820* and its orthologs across various *Mycobacterium* species. Sequences from *M. tuberculosis*, *M. smegmatis*, *M. leprae*, *M. marinum*, and *M. bovis* were aligned using ClustalW and visualized with ESPript 3.0. **(B)** Transmembrane topology prediction of *Rv0820* (left) and *Ms_5779* (right). **(C)** Signal peptide probability analysis of *Rv0820* (left) and *Ms_5779* (right).

To establish the functional relevance of *Ms_5779* as a surrogate for *Rv0820*, we first performed a comprehensive bioinformatic characterization of both proteins. *Rv0820* and *Ms_5779* each encode proteins of 258 amino acids, with the corresponding open reading frames spanning 777 bp. Sequence analysis indicates that both proteins possess conserved sequence motifs characteristic of the ATP-binding cassette (ABC) transporter superfamily, providing the basis for their annotation. Multiple sequence alignment using ClustalW revealed high amino acid sequence identity between *Rv0820* and *Ms_5779*, with conserved residues distributed throughout the full length of both proteins (Figure 1A), strongly supporting the use of *Ms_5779* as a functional proxy for *Rv0820* in *M. smegmatis*. Importantly, conserved domain analysis and multiple sequence alignment confirmed that both Rv0820 and *Ms_5779* possess canonical ATP-binding cassette (ABC) domains characteristic of known ABC transporter nucleotide-binding subunits. Specifically, the alignment revealed the presence of the highly conserved Walker A motif (GXXGXGKS/T, typically responsible for ATP binding), the universal ABC signature motif (LSGGQ, unique to the ABC transporter superfamily), and the Walker B motif (hhhhDE, required for ATP hydrolysis) within their sequences. The presence of these hallmark motifs provides definitive structural homology evidence classifying *Ms_5779*/Rv0820 within the ABC transporter superfamily, despite their lack of transmembrane domains.

Physicochemical analysis using the ProtParam tool (ExPASy) revealed that *Ms_5779* has an aliphatic index of 95.97, suggesting high thermostability, and a grand average of hydropathicity (GRAVY) value of −0.152, indicating a slightly hydrophilic nature. Transmembrane topology prediction using TMHMM Server v2.0 revealed that neither *Ms_5779* nor *Rv0820* contains canonical transmembrane helices (Figure 1B), a feature atypical of classical efflux pump proteins that depend on transmembrane domains for substrate translocation. Signal peptide analysis using SignalP-5.0 predicted standard signal peptide probabilities of 2.70% for *Ms_5779* and 2.20% for *Rv0820*, with lipoprotein signal peptide probabilities of 1.62 and 3.75%, respectively (Figure 1C). The collective absence of transmembrane domains and signal peptides is consistent with a cytoplasmic ATP-binding subunit function, as seen in PstB-type ATPases of ABC phosphate importers, where the ATPase subunit is inherently cytoplasmic and lacks transmembrane helices. This architecture does not exclude transporter function but argues against *Ms_5779* being an integral membrane efflux pump. Whether *Ms_5779* functions as part of a multi-component phosphate transport complex or serves an alternative structural role in the cell envelope requires experimental determination beyond bioinformatic prediction.

### Construction and validation of a CRISPRi system for transcriptional silencing of *Ms_5779* in *Mycobacterium smegmatis*

To functionally characterize *Ms_5779*, we constructed a two-plasmid CRISPRi system in *M. smegmatis* mc^2^-155. The system comprises pRH2502, which constitutively expresses the catalytically inactive dCas9 protein (kanamycin resistance), and pRH2521, which carries the sgRNA expression cassette (hygromycin resistance). Two sgRNAs were designed to target the *Ms_5779* coding sequence at positions +57 (*Ms_5779*+57) and +162 (*Ms_5779*+162) relative to the transcription start site, selected on the basis of high on-target scores and minimal predicted off-target activity as assessed by Benchling software. Additionally, a non-targeting control sgRNA (sgRNA-NC) targeting a potato (*Solanum tuberosum*) SPUD sequence was incorporated. Since this sequence lacks homology to the *M. smegmatis* genome, it serves as a robust control to account for any potential sequence-independent toxicity or off-target effects associated with the dCas9-sgRNA complex (Table 1).

**Table 1:** Oligonucleotides used for the construction of CRISPRi sgRNA expression plasmids.

Oligonucleotide Name	Sequence (5′ to 3′)
sgRNA-5779-57-F	CCGCGAGTGACACGTCGGTA
sgRNA-5779-57-R	TACCGACGTGTCACTCGCGG
sgRNA-5779-162-F	CACCGCGAGTGACACGTCGG
sgRNA-5779-162-R	CCGACGTGTCACTCGCGGTG
sgRNA-NC-F	TTGTGCACACTCAAAATTGG
sgRNA-NC-R	CCAATTTTGAGTGTGCACAA

All oligonucleotide sequences are shown in the 5′ to 3′ direction. *Ms_5779* target positions (+57 and +162) are relative to the transcription start site. The non-targeting control (NC) oligonucleotides were designed based on a specific sequence from the potato SPUD gene, which lacks homology to the *M. smegmatis* genome, to serve as a negative control for CRISPRi sequence-independent effects.

**Figure 2:**
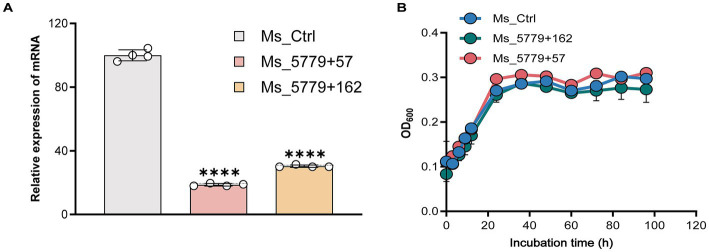
Construction and validation of a CRISPRi system for transcriptional silencing of *Ms_5779* in *M. smegmatis*. **(A)** Transcriptional levels of *Ms_5779* determined by RT-qPCR. **(B)** Growth kinetics of the silenced and control strains under standard culture conditions. ****, *p* < 0.0001 vs. *Ms_Ctrl.*

The CRISPRi plasmids were constructed and transformed into *M. smegmatis* as described in Methods, generating the final silenced and control strains. RT-qPCR analysis demonstrated that both sgRNAs effectively repressed *Ms_5779* transcription (Figure 2A). The *Ms_5779*+57 strain achieved 81.33% repression and the *Ms_5779*+162 strain achieved 69.67% repression relative to non-targeting control strain (Ms_Ctrl). The superior repression efficiency of the +57 sgRNA is consistent with the established principle that sgRNAs targeting positions proximal to the transcription start site generally achieve stronger CRISPRi-mediated repression (Wong and Rock, 2021; Zhang et al., 2021). Although the difference in repression efficiency between the two sgRNAs (81% vs. 69%, a ~12 percentage point difference) may appear modest, this difference was statistically significant (*p* < 0.001, Student’s *t*-test) and was sufficient to produce measurable differences in multiple phenotypic readouts. The concordant direction of phenotypic effects across both silenced strains, despite their different repression levels, provides internal validation of the specificity of the *Ms_5779* knockdown phenotype. Neither silenced strain exhibited altered growth kinetics relative to the Ms_Ctrl under standard culture conditions, confirming that the observed phenotypes in subsequent assays are attributable to *Ms_5779* repression rather than growth impairment (Figure 2B). These results confirm the successful establishment of a CRISPRi system yielding two silenced strains with distinct repression levels suitable for comparative functional analysis.

### *Ms_5779* silencing confers broad-spectrum drug hypersusceptibility, most pronounced against fluoroquinolones

To determine whether *Ms_5779* contributes to drug tolerance, we measured the MICs of six clinically relevant anti-TB drugs as described in Methods. *Ms_5779* silencing resulted in significantly increased susceptibility to all tested drugs (Table 2). The most pronounced effect was observed for norfloxacin (NOR), a fluoroquinolone: the MIC decreased from 1 μg/mL in the control to 0.03125 μg/mL in *Ms_5779*+57 (32-fold reduction) and 0.125 μg/mL in *Ms_5779*+162 (8-fold reduction). Substantial MIC reductions were also observed for rifampicin (RIF; 16-fold in *Ms_5779*+57, 32-fold in *Ms_5779*+162) and isoniazid (INH; 16-fold in both strains). The relationship between repression depth and drug susceptibility was drug-class-dependent: *Ms_5779*+57 exhibited greater susceptibility to fluoroquinolones (NOR: 32-fold vs. 8-fold; MXF: 8-fold vs. 4-fold), whereas *Ms_5779*+162 showed greater susceptibility to RIF (32-fold vs. 16-fold) and capreomycin (CPM: 16-fold vs. 4-fold), with equivalent effects for INH and streptomycin (SM; 16-fold and 4-fold, respectively). This drug-class-dependent pattern indicates that distinct aspects of cell wall integrity, each differentially sensitive to the degree of *Ms_5779* depletion, differentially impact bacterial susceptibility to distinct antibiotic classes, likely reflecting a complex interplay between altered barrier permeability and synergistic envelope-associated vulnerabilities.

**Table 2:** MICs of anti-tuberculosis drugs against CRISPRi-silenced strains.

Strain	Rifampicin (RIF)		Isoniazid (INH)		Streptomycin (SM)		Capreomycin (CPM)		Moxifloxacin (MXF)		Norfloxacin (NOR)		MIC (μg/mL)	FC	MIC (μg/mL)	FC	MIC (μg/mL)	FC	MIC (μg/mL)	FC	MIC (μg/mL)	FC	MIC (μg/mL)	FC
Ms_Ctrl	4	—	4	—	4	—	2	—	0.125	—	1	—
*Ms_5779*+57	0.25	**16**	0.25	**16**	1	4	0.5	4	0.015625	8	0.03125	**32**
*Ms_5779*+162	0.125	**32**	0.25	**16**	1	4	0.125	**16**	0.03125	4	0.125	8

MIC, minimum inhibitory concentration determined by broth microdilution. FC, fold change = MICcontrol / MICstrain. —, reference strain. Bold values indicate FC ≥ 16. RIF, rifampicin; INH, isoniazid; SM, streptomycin; CPM, capreomycin; MXF, moxifloxacin; NOR, norfloxacin.

**Figure 3:**
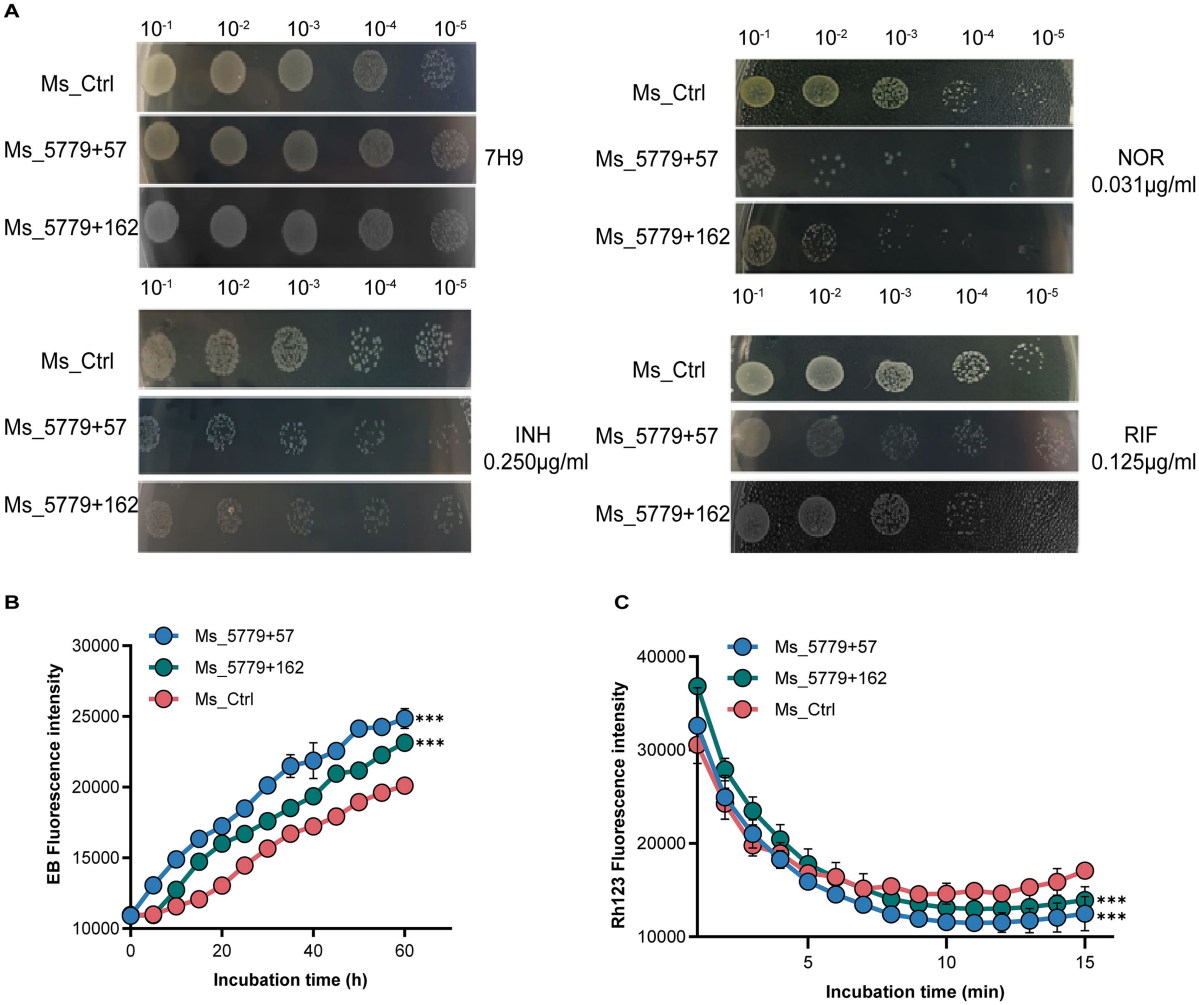
*Ms_5779* silencing increases drug susceptibility and alters cell wall permeability. **(A)** Spot assay (10-fold serial dilution). *Ms_Ctrl*, *Ms_5779+57*, and *Ms_5779+162* strains were inoculated on Middlebrook 7H10 agar supplemented with indicated concentrations of RIF, INH, or NOR. **(B)** Ethidium bromide (EB) accumulation assay. Intracellular fluorescence intensity was measured over 60 min in the presence of 1 μg/mL EB. **(C)** Membrane potential measurement using rhodamine 123 over 15 min. ***, *p* < 0.001 *vs. Ms_Ctrl.*

Serial dilution spot assays on drug-supplemented 7H10 agar corroborated the MIC data and provided visual confirmation of differential drug susceptibility (Figure 3A). Ethidium bromide (EB) accumulation assays, performed as described in Methods, demonstrated that *Ms_5779*+57 bacteria exhibited significantly elevated intracellular EB fluorescence relative to the Ms_Ctrl control, indicating increased intracellular accumulation of ethidium bromide upon *Ms_5779* silencing. This accumulation could reflect increased passive permeability of the cell envelope to hydrophilic compounds, reduced active efflux of ethidium bromide, or a combination of both mechanisms. Given that *Ms_5779* lacks canonical transmembrane helices and is thereforeunlikely to function as a direct efflux transporter, increased passive permeability is the more parsimonious interpretation (Figure 3B). Membrane potential measurements using rhodamine 123 revealed altered electrochemical gradient dynamics in silenced strains compared to the control (Figure 3C).

### *Ms_5779* is required for biofilm formation in *Mycobacterium smegmatis*

**Figure 4:**
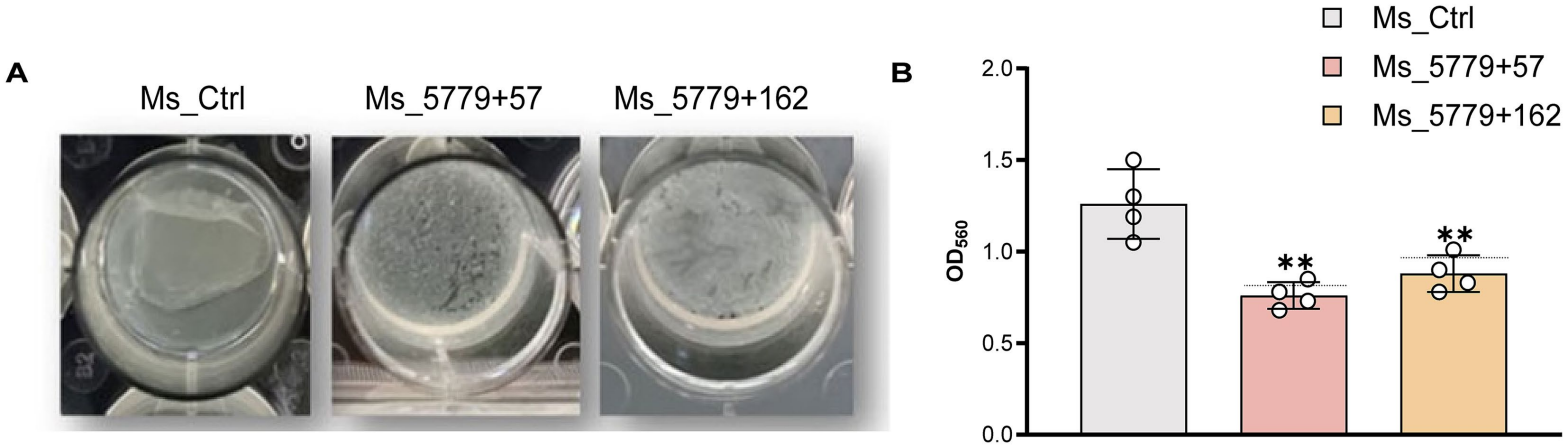
*Ms_5779* silencing impairs biofilm formation in *M. smegmatis*. **(A)** Representative macroscopic images of biofilms formed by *Ms_Ctrl*, *Ms_5779+57*, and *Ms_5779+162* strains in 12-well plates after 5 days of static incubation in Middlebrook 7H9 broth at 37 °C. **(B)** Quantitative crystal violet biofilm assay. **, *p* < 0.01 vs. Ms_Ctrl.

Biofilm formation assays, performed as described in Methods, revealed that *Ms_5779* silencing markedly impaired biofilm formation. Macroscopic examination demonstrated that the Ms_Ctrl formed a continuous, structurally intact biofilm pellicle at the air–liquid interface, whereas *Ms_5779*+57 and *Ms_5779*+162 strains produced fragmented, discontinuous biofilms with visible structural defects (Figure 4A). Quantitative crystal violet assays confirmed significantly reduced biofilm biomass in both silenced strains relative to the control, with *Ms_5779*+57 exhibiting greater impairment than *Ms_5779*+162 (Figure 4B), demonstrating a dose-dependent biofilm deficiency. These results indicate that *Ms_5779* is required for normal biofilm formation in *M. smegmatis*. The mechanism by which *Ms_5779* depletion impairs biofilm formation is not established by the present data; possibilities include altered cell surface hydrophobicity, changes in extracellular matrix component secretion, or reduced cell–cell adhesion secondary to cell envelope disruption.

### *Ms_5779* silencing impairs bacterial survival under oxidative, acidic, and thermal stress

**Figure 5:**
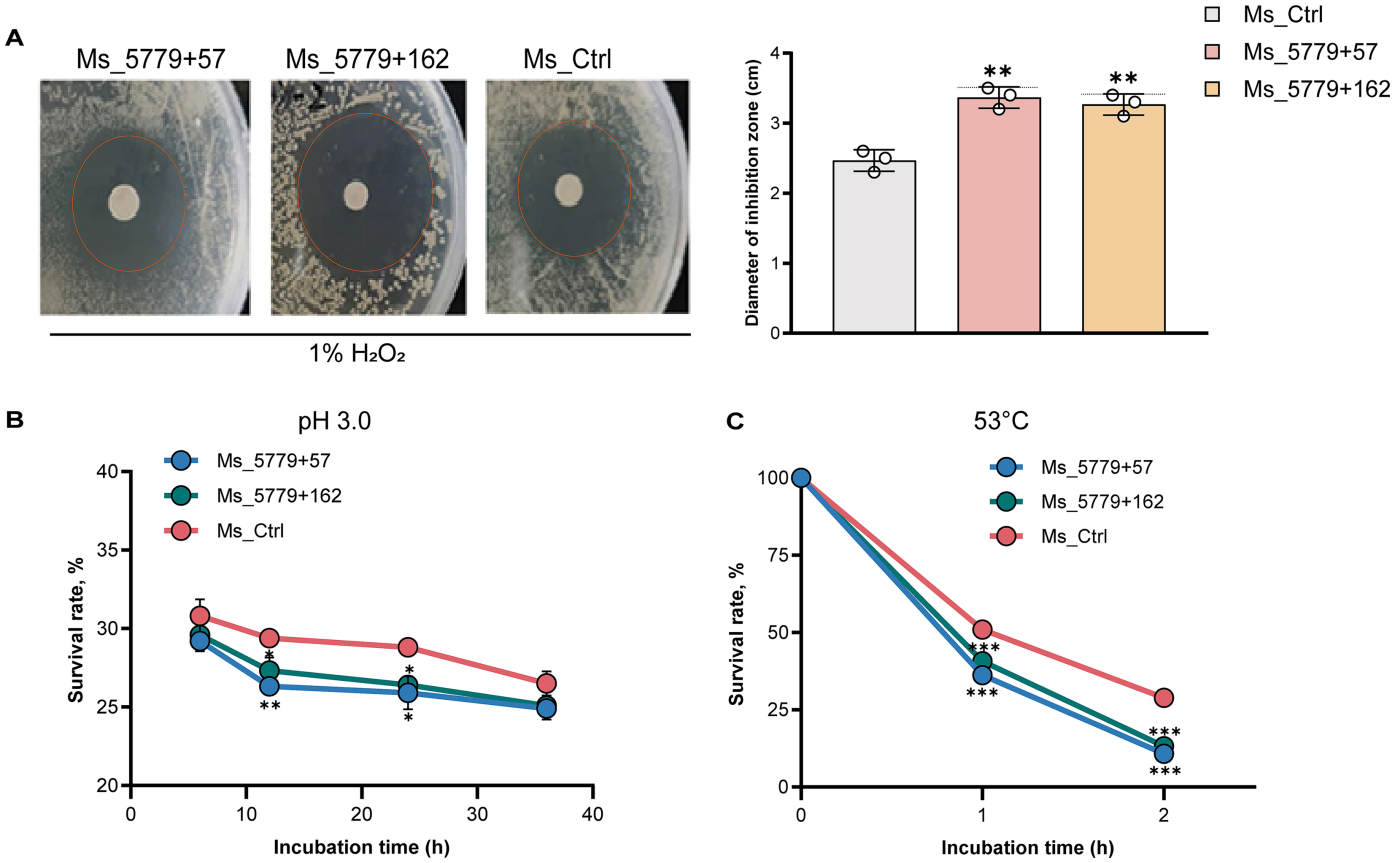
*Ms_5779* silencing reduces bacterial survival under oxidative, acidic, and heat stress. **(A)** Representative images of the H_2_O_2_ disk diffusion assay. **(B)** Survival rate of *Ms_Ctrl*, *Ms_5779+57*, and *Ms_5779+162* strains under acidic stress (pH 3.0). **(C)** Survival rate of *Ms_Ctrl*, *Ms_5779+57*, and *Ms_5779+162* strains under heat stress (53 °C). *, *p* < 0.05 vs. *Ms_Ctrl*. **, *p* < 0.01 vs. Ms_Ctrl. ***, *p* < 0.001 vs. Ms_Ctrl.

Stress assays, performed as described in methods. Disk diffusion assays using 1% H₂O₂ demonstrated that both *Ms_5779*+57 and *Ms_5779*+162 strains formed significantly larger inhibition zones than the Ms_Ctrl, with *Ms_5779*+57 exhibiting the largest zone (Figure 5A), indicating heightened susceptibility to oxidative stress. Survival assays revealed that both silenced strains exhibited significantly reduced viability relative to the Ms_Ctrl control by 12 h, with *Ms_5779*+57 showing the lowest survival rate (Figure 5B). Heat stress experiments at 53 °C demonstrated that *Ms_5779* silencing significantly impaired thermotolerance, with both silenced strains exhibiting markedly lower survival than the control after 1 h of heat exposure (Figure 5C).

### *Ms_5779* silencing attenuates intracellular survival and virulence-associated phenotypes in macrophages

**Figure 6:**
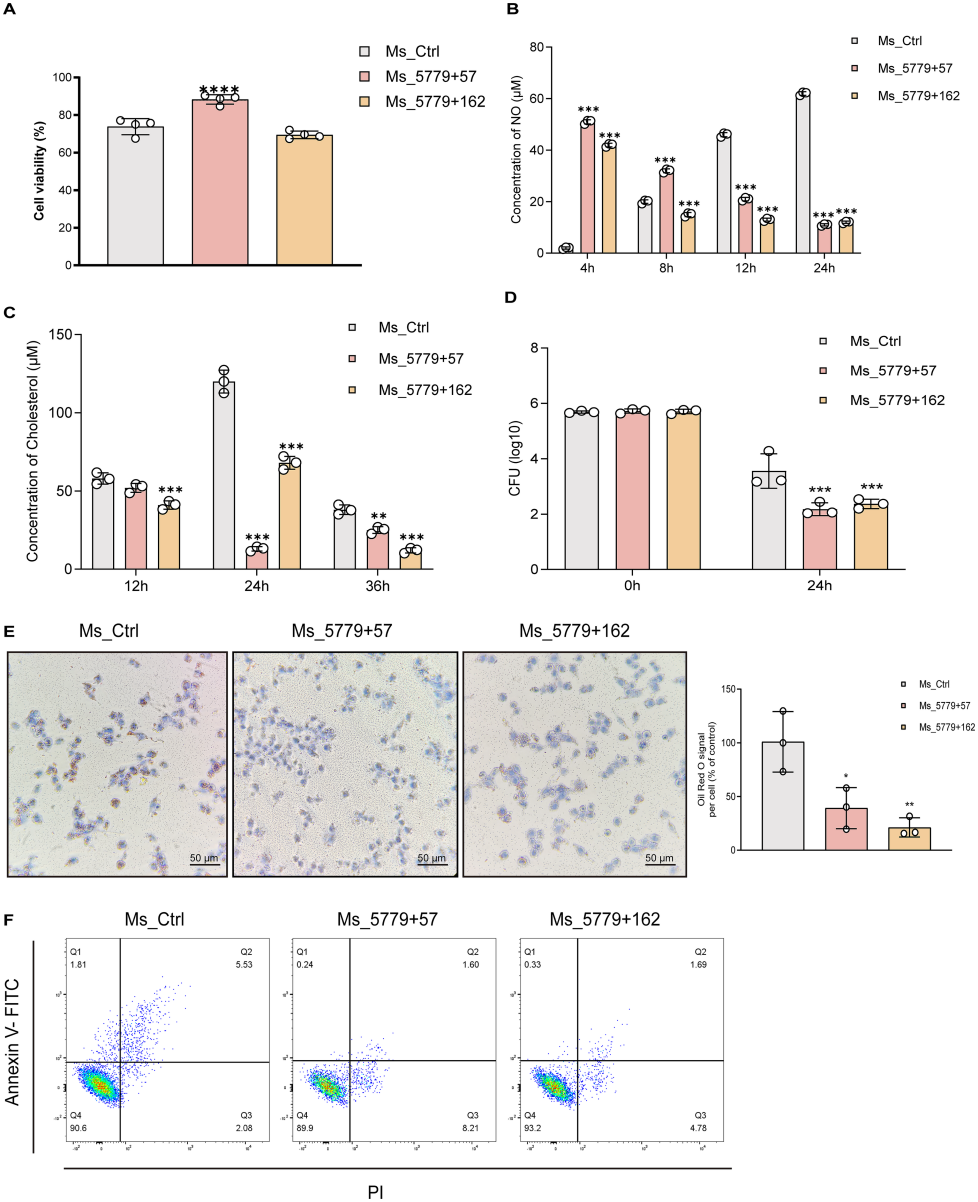
*Ms_5779* silencing attenuates intracellular survival and pathogenicity in RAW264.7 macrophages. **(A)** Macrophage viability assessed by CCK-8 assay. **(B)** Nitric oxide (NO) production in macrophages measured by the Griess reagent method. **(C)** Cholesterol content in infected macrophages. **(D)** Intracellular bacterial survival within RAW264.7 macrophages. Macrophages were infected with the *Ms_Ctrl*, *Ms_5779+162*, and *Ms_5779+57* strains, and intracellular bacterial loads were determined by CFU enumeration at 0 and 24 h post-infection. **(E)** Representative Oil Red O staining images of macrophages at 24 h post-infection. Scale bar, 50 μm. Quantification of Oil Red O signal per cell (IntDen/cell count, normalized to *Ms_Ctrl*). **(F)** Quantification of total apoptosis rates from flow cytometric analysis. **, *p* < 0.01 vs. *Ms_Ctrl*. ***, *p* < 0.001 vs. *Ms_Ctrl*. ****, *p* < 0.0001 vs. *Ms_Ctrl*.

Macrophage infection assays were performed as described in Methods. Macrophages infected with the Ms_Ctrl exhibited progressively declining cell viability, consistent with ongoing intracellular bacterial replication and cytotoxicity, whereas macrophages infected with *Ms_5779*+57 maintained significantly higher viability throughout the observation period (Figure 6A), indicating substantially reduced intracellular bacterial burden. NO production in macrophages infected with *Ms_5779*+57 and *Ms_5779*+162 strains was markedly elevated at 4 h post-infection but declined progressively thereafter, reaching levels below those of the Ms_Ctrl by 12–24 h (Figure 6B). In contrast, macrophages infected with the Ms_Ctrl showed sustained and progressive increases in NO production throughout the 24-h observation period. Intracellular cholesterol measurements revealed markedly elevated cholesterol levels in macrophages infected with the Ms_Ctrl at 24 h post-infection, indicative of active cholesterol acquisition by intracellular bacteria; macrophages infected with *Ms_5779*+57 showed only a transient increase that returned to near-baseline levels by 36 h (Figure 6C). To directly confirm the reduced intracellular bacterial burden inferred from these host cell readouts, we quantified intracellular survival using CFU enumeration. Initial bacterial loads at 0 h post-infection were comparable across all groups, indicating that *Ms_5779* silencing does not affect the initial phagocytosis by macrophages. However, by 24 h post-infection, the intracellular survival of the silenced strains (*Ms_5779*+162 and *Ms_5779*+57) was profoundly impaired compared to the Ms_Ctrl strain (Figure 6D). Oil Red O staining revealed a marked reduction in neutral lipid accumulation within macrophages infected with the *Ms_5779*+57 and *Ms_5779*+162 strains, as compared to those infected with the Ms_Ctrl strain (Figure 6E). Quantitative analysis shows that macrophages infected with *Ms_5779*+57 and *Ms_5779*+162 exhibit significantly reduced lipid accumulation compared to those infected with Ms_Ctrl.

Flow cytometric analysis of Annexin V/PI-stained cells revealed that while the overall apoptosis rates were relatively low, infection with the Ms_Ctrl strain induced a modest increase in late-stage macrophage apoptosis (Q2) compared to the silenced strains. This apoptotic response was attenuated in macrophages infected with *Ms_5779*+162 and further reduced with *Ms_5779*+57 (Figure 6F). The decreased capacity of the *Ms_5779* silenced strains to induce apoptosis suggests that *Ms_5779* may play a partial role in facilitating *M. smegmatis*-induced cell death.

## Discussion

The central finding of this study is that *Ms_5779*/*Rv0820*, despite its annotation as an ABC transporter, appears to function primarily as a critical factor indispensable for cell envelope integrity, exhibiting a functional profile distinct from a classical drug efflux pump. The structural basis for this inference is straightforward: *Rv0820* lacks canonical transmembrane helices, making conventional membrane-spanning substrate translocation mechanistically implausible. The functional data are consistent with this model. *Ms_5779* silencing increased cell wall permeability to hydrophilic compounds, as demonstrated by ethidium bromide accumulation assays, and disrupted the transmembrane electrochemical gradient, as shown by rhodamine 123 measurements. Critically, the resulting drug hypersusceptibility was broad-spectrum rather than substrate-specific (a pattern more consistent with generalized barrier disruption than with the loss of a dedicated efflux transporter, which would be expected to confer selective resistance to its cognate substrates). The convergence of drug hypersusceptibility, biofilm defects, multi-stress sensitivity, and attenuated macrophage virulence in *Ms_5779*-silenced bacteria further argues that the cell wall structural role of this protein is not peripheral but central to mycobacterial fitness across multiple host-relevant environments. Whether *Ms_5779*/*Rv0820* retains any residual transport activity remains to be determined by direct biochemical characterization; the present data strongly suggest that its dominant physiological function is maintenance of cell wall barrier integrity.

Although compromised cell envelope integrity was observed following the silencing of *Ms_5779*, this phenotype may not stem from a direct role of *Ms_5779* within the envelope. An alternative explanation is that the disruption of the Pst system leads to alterations in intracellular phosphate homeostasis. Phosphate homeostasis is critical for bacterial cellular processes; its imbalance can trigger metabolic remodeling, which subsequently affects the biosynthesis and maintenance of cell surface components (Lamarche et al., 2008). Consequently, the cell envelope defects observed in the *Ms_5779*-silenced strain are likely the result of inhibited phosphate metabolism, rather than the direct loss of a structural scaffold. This disruption of phosphate homeostasis indirectly induces the phenotypic alterations in the cell envelope. However, we acknowledge that direct measurements of intracellular phosphate levels in the silenced strain, along with complementation experiments utilizing the intact pst operon, are necessary to formally distinguish between these models. This represents a key priority for future research.

Four independent lines of evidence converge on the cell wall structural model. First, ethidium bromide accumulation assays demonstrated that the silencing of *Ms_5779* enhanced the intracellular accumulation of hydrophilic compounds. This increased accumulation could potentially be attributed to enhanced cell envelope permeability, impaired active efflux of EB, or a combination of both factors. Given the absence of typical transmembrane helices, *Ms_5779* is structurally unlikely to function directly as an efflux transporter; thus, an increase in passive permeability constitutes the most plausible explanation. Nevertheless, we must acknowledge the inherent limitations of the EB accumulation assay. In the absence of complementary experiments, the possibility of altered active efflux activity cannot be definitively precluded. Second, membrane potential measurements revealed altered electrochemical gradients in silenced strains, consistent with perturbation of the cell wall-associated proton motive force that depends on an intact envelope. Third, the impaired biofilm formation in silenced strains implicates *Ms_5779* in the assembly or maintenance of the extracellular matrix, which is built on and anchored to cell wall components. Fourth, the broad-spectrum stress hypersensitivity spanning oxidative, acidic, and thermal insults is the hallmark of a generalized envelope defect, not the loss of a substrate-specific pump. Therefore, the present data support the notion that *Ms_5779* serves as a multifunctional cell wall component, which plays a role in tolerance to diverse physicochemical stresses. This functional profile places *Ms_5779*/*Rv0820* in a conceptual category occupied by the MmpL family of RND-type transporters, several of which contribute to cell wall lipid biosynthesis and structural integrity rather than, or in addition to, direct drug efflux (Yamamoto et al., 2021). While *M. abscessus* differs from *M. smegmatis* in outer membrane lipid composition, the general principle that membrane permeability and efflux mechanisms act synergistically to govern intrinsic antibiotic resistance—a foundational paradigm in mycobacterial physiology(Nguyen and Thompson, 2006)—has been elegantly demonstrated in *M. abscessus* (McGowen et al., 2025), is reinforced by studies of mmpL3 (Yuliani et al., 2024) and cwlM (Yang et al., 2025) in *M. smegmatis*, which directly link cell wall integrity to antibiotic susceptibility.

The disproportionate fluoroquinolone hypersusceptibility (32-fold MIC reduction for norfloxacin, versus 4–16-fold for other drug classes) deserves mechanistic attention. The mycobacterial cell wall is an exceptionally low-permeability barrier: its outer leaflet, dominated by mycolic acids esterified to arabinogalactan and intercalated with extractable lipids, creates a hydrophobic shield that is particularly effective against hydrophilic zwitterionic compounds such as fluoroquinolones (Jacobo-Delgado et al., 2023). This architecture means that fluoroquinolone accumulation is exquisitely sensitive to even modest increases in envelope permeability, the depletion of PhoT (*Ms_5779*) induces structural disorganization and reduced hydrophobicity within this lipid layer, permitting an exponential increase in the passive influx of these drugs. This mechanistically explains why the same degree of *Ms_5779* depletion that produces a 4-fold streptomycin MIC shift produces a 32-fold norfloxacin shift. The clinical weight of this observation is considerable: pre-XDR-TB, defined by MDR/RR-TB with additional fluoroquinolone resistance, already accounts for 19% of the global MDR/RR-TB burden (World Health Organization, 2025), and fluoroquinolones remain the backbone of second-line regimens. Whole-genome sequencing of resistant clinical isolates has identified envelope-associated gene variants as contributors to fluoroquinolone resistance (Alvarado-Peña et al., 2025; Chimal-Muñoz et al., 2025), and efflux pump inhibitors can partially restore fluoroquinolone susceptibility in resistant strains (Rodrigues et al., 2020), consistent with the envelope permeability model. Notably, *Ms_5779*-silenced bacteria also showed reduced foamy macrophage induction, suggesting that the same cell wall disruption that sensitizes bacteria to fluoroquinolones also impairs intracellular lipid exploitation. This co-vulnerability raises the possibility that *Rv0820*-targeting strategies could synergize with fluoroquinolone-based regimens by simultaneously compromising the permeability barrier and the intracellular niche.

The macrophage data extend the cell wall structural model from the drug tolerance context into the host–pathogen interface. Five virulence-associated phenotypes were coordinately attenuated by *Ms_5779* silencing: macrophage cytotoxicity, nitric oxide production, intracellular cholesterol exploitation, foamy macrophage induction, and apoptosis. This co-attenuation is most parsimoniously explained by a single upstream defect, namely a structurally compromised cell wall, rather than five independent functional losses. The mycobacterial cell envelope is the primary molecular interface with the host: LAM, PIMs, and mycolic acids serve as ligands for macrophage pattern recognition receptors (Chandra et al., 2022; Bo et al., 2023), and their correct presentation depends on an intact envelope architecture. Structural disruption of the cell wall would simultaneously impair phagosomal escape, alter TLR/CLR signalling, and increase susceptibility to phagolysosomal antimicrobial effectors, a cascade that would account for all five attenuated phenotypes, we further propose that this phenotypic alteration may stem from impaired presentation of critical virulence lipids on the bacterial surface to macrophage receptors following disruption of cell wall integrity, thereby compromising the bacterial ability to evade lysosomal killing and modulate host cholesterol metabolism. The NO kinetics in macrophages infected with silenced strains are consistent with this model. The transient NO elevation peaking at 4 h post-infection reflects the initial innate immune response to bacterial invasion; the subsequent progressive decline to below-control levels by 12–24 h indicates efficient bacterial clearance: once the structurally compromised bacteria are eliminated, the innate immune stimulus is removed and NO production subsides. In contrast, the sustained and progressive NO elevation in Ms_Ctrl-infected macrophages reflects persistent bacterial survival and ongoing innate immune activation. This divergent NO kinetic pattern thus provides independent temporal evidence for the more efficient clearance of *Ms_5779*-silenced bacteria. The cholesterol exploitation defect is particularly informative. Cholesterol is an essential intracellular carbon and energy source for Mtb (Pawełczyk et al., 2021; Chen Z. et al., 2024), and its import depends on the Mce4 lipid transport system, which requires an intact outer membrane for substrate capture. Recent work has further shown that cholesterol metabolism is integrated with pH sensing and potassium homeostasis in the phagosome (Chen Y. et al., 2024; Chen et al., 2025), suggesting that cell wall integrity coordinates multiple aspects of intracellular metabolic adaptation. The downstream consequences, including reduced foamy macrophage formation and attenuated apoptosis induction (Bedard et al., 2023; Rombouts and Neyrolles, 2023; Simwela et al., 2025), reinforce the view that *Ms_5779* sits at the apex of a cell wall-dependent virulence programme. These observations are further consistent with reports that Mtb virulence lipids such as PDIM inhibit autophagy to promote intracellular survival (Mittal et al., 2024), and that the spatial positioning of Mtb within macrophages, which is itself dependent on cell wall properties, determines drug tolerance and immune evasion capacity (Sahu et al., 2025).

By deploying two sgRNAs targeting positions +57 and +162 within *Ms_5779*, we generated strains with 81 and 69% repression respectively, a deliberate titration that revealed drug-class-dependent dose–response relationships invisible to a binary knockout approach. The deeper-silenced *Ms_5779*+57 strain showed greater fluoroquinolone hypersusceptibility, while *Ms_5779*+162 showed greater susceptibility to rifampicin and capreomycin. This divergence implies that distinct structural features of the cell wall, each differentially sensitive to the degree of *Ms_5779* depletion, govern permeability to different antibiotic classes, a nuance that would have been missed with a single silencing depth. Although the absolute difference in repression between the two strains is approximately 12%, this narrow transcriptional dynamic range translates to a ~38.5% relative depletion in the residual transcript pool (30.33% vs. 18.67% remaining transcripts). The exponentially amplified phenotypic differences observed—such as the transition from an 8-fold to a 32-fold reduction in norfloxacin MIC—strongly suggest a non-linear threshold effect. It appears that dropping below a critical ~20% transcript threshold triggers a disproportionate structural collapse of the envelope permeability barrier, highlighting the profound phenotypic vulnerability of this specific structural determinant. Indeed, the superior repression efficiency of the +57 sgRNA is consistent with the established principle that sgRNAs targeting positions proximal to the transcription start site generally achieve stronger CRISPRi-mediated repression(Wong and Rock, 2021; Zhang et al., 2021), and the absence of growth defects in either silenced strain confirms that the phenotypes reflect *Ms_5779* function rather than non-specific fitness costs. More broadly, this study illustrates the power of CRISPRi-based functional genomics for interrogating the ~30 uncharacterized ABC transporters in Mtb: genome-wide CRISPRi screens have already identified novel drug resistance determinants and essential gene vulnerabilities (Winkler et al., 2023), and the macrophage-compatible format demonstrated here enables functional characterization under physiologically relevant infection conditions. The approach is particularly well-suited to structurally atypical proteins like *Rv0820*, where sequence-based functional prediction fails and experimental interrogation is the only path to resolution.

The choice of *M. smegmatis* mc^2^-155 as the experimental system is justified on both practical and scientific grounds, but its limitations must be acknowledged alongside its strengths. Practically, *M. smegmatis* offers BSL-1 containment, a 3–4 h generation time versus 18–24 h for Mtb, and transformation efficiencies orders of magnitude higher, enabling the genetic manipulations and phenotypic screens that would be prohibitively slow or technically intractable in Mtb (Shiloh and Champion, 2010; Sparks et al., 2023). Scientifically, the high sequence conservation between *Ms_5779* and *Rv0820* provides strong *a priori* justification for functional inference, a principle validated repeatedly: cell wall biosynthesis genes, efflux pumps, and stress response factors characterized first in *M. smegmatis* have been confirmed in Mtb (Nguyen and Thompson, 2006), and the RAW264.7 macrophage infection model has been successfully employed with *M. smegmatis* to investigate virulence-associated phenotypes (Cheung et al., 2022). The critical caveat is that *M. smegmatis* and Mtb differ in outer cell wall lipid composition, the repertoire of Mtb-specific virulence factors, and the complexity of the intracellular environment during human infection. The functional conclusions drawn here are therefore best understood as mechanistic hypotheses that require direct validation in Mtb, a necessary next step that the present study is designed to motivate and inform.

Four limitations define the agenda for follow-up work. First, the biochemical substrate(s) of *Ms_5779*/*Rv0820* remain unidentified. The absence of transmembrane helices argues against classical substrate translocation, but whether the protein has any residual transport activity, perhaps for a non-lipid substrate, cannot be excluded without *in vitro* reconstitution assays using purified protein in proteoliposomes. Second, and most critically, the definitive subcellular localization of *Ms_5779*/Rv0820 remains uncharacterized. We explicitly acknowledge that our current extensive phenotypic assays (MIC, EtBr uptake, membrane potential, and multi-stress survival) provide strong correlative evidence of a compromised cell envelope, but do not offer direct biochemical proof of a cell wall localization. Given the absence of transmembrane helices and signal peptides, the protein could plausibly reside in the cytoplasm, perhaps functioning as an NBD that exerts systemic effects on cell wall homeostasis. To rigorously resolve this paradox, future experimental directions utilizing subcellular fractionation (separating the cytosol, plasma membrane, and cell wall portions) combined with immunoblotting, alongside advanced proximity labeling (APEX2) or fluorescent microscopy of GFP-tagged variants, are urgently required to unambiguously define its physical localization. Although the CRISPRi system employed in this study yielded consistent and dose-dependent phenotypes across two distinct sgRNAs—thereby robustly substantiating the specific function of *Ms_5779*—the inherent limitations of this technology must be acknowledged. Specifically, the transcriptional repression mediated by the dCas9-sgRNA complex may elicit polar effects on downstream genes located within the same operon. While the gradient phenotypes resulting from varying degrees of knockdown alleviate this concern to some extent, completely decoupling the genuine function of *Ms_5779* from potential downstream interference necessitates the construction of precise gene deletion mutants. Consequently, future research efforts will be directed towards generating knockout and complemented strains of this gene to further elucidate the molecular mechanisms governing this cell envelope determinant. The structural basis of the cell wall-associated function is unknown; cryo-EM or X-ray crystallography, which recently resolved the Rv1217c–Rv1218c rifampicin efflux complex (Wang et al., 2024), would provide the mechanistic resolution needed to understand how a protein without transmembrane helices contributes to envelope architecture. Third, the transcriptional regulation of *Ms_5779*/*Rv0820* under drug stress and host infection conditions has not been investigated; identifying the regulatory circuits controlling this locus could reveal inducible vulnerabilities amenable to therapeutic exploitation. Fourth, and most critically, the essentiality of *Rv0820* in Mtb has not been assessed; CRISPRi-based conditional knockdown in Mtb under both *in vitro* and macrophage infection conditions is the essential next experiment.

In conclusion, this study addresses the structural paradox of *Rv0820* and provides strong evidence to recast it as a cell wall structural determinant rather than a conventional efflux pump. The functional consequences of its loss are broad and clinically relevant: drug hypersusceptibility across six antibiotic classes, with a 32-fold fluoroquinolone effect that is particularly timely given the pre-XDR-TB crisis; impaired biofilm formation and multi-stress tolerance; and attenuated intracellular virulence in macrophages. These phenotypes collectively argue that *Rv0820* is not a peripheral annotation artifact but a functionally important node in mycobacterial cell wall biology. Targeting a structural scaffold protein rather than a specific efflux pump offers a mechanistically distinct therapeutic strategy, one that compromises the entire permeability barrier rather than a single transport pathway.

## Data Availability

The original contributions presented in the study are included in the article/supplementary material, further inquiries can be directed to the corresponding authors.

## Footnotes

1
https://mycobrowser.epfl.ch/

2
https://www.ebi.ac.uk/Tools/msa/clustalw2/

3
https://espript.ibcp.fr/

4
https://web.expasy.org/protparam/

5
https://services.healthtech.dtu.dk/service.php?SignalP-5.0

6
https://services.healthtech.dtu.dk/service.php?TMHMM-2.0

7
https://www.benchling.com/
